# Elaboration of fibrous structured activated carbon from olive pomace via chemical activation and low-temperature pyrolysis

**DOI:** 10.1016/j.heliyon.2024.e38886

**Published:** 2024-10-02

**Authors:** Imad Alouiz, Mouhssine Benhadj, Elmontassir Dahmane, Mohamed Sennoune, Mohamed-Yassine Amarouch, Driss Mazouzi

**Affiliations:** R.N.E Laboratory, Multidisciplinary Faculty of Taza, University Sidi Mohamed Ben Abdellah, Fez, Morocco

**Keywords:** Olive pomace, Chemical activation, Pyrolysis, Activated carbon, Fibrous structure

## Abstract

The objective of this study is to examine the preparation of activated carbon with a fibrous structure obtained from olive pomace through a chemical activation process using phosphoric acid (H_3_PO_4_) as the activating agent under air at a lower temperature. According to the findings, the most effective conditions to achieve high-performance activated carbon were 22 vol% of H_3_PO_4_, a 2-h chemical activation impregnation residence time at 50 °C, and a 500 °C pyrolysis temperature for 1 h. Structural analysis revealed that activated carbons possess highly developed textural and structural properties, resulting in an iodine value of 923 mg/g and a specific surface of 1400 m^2^/g. In addition to its microporosity, the produced carbon exhibits a highly developed fibrous structure, providing excellent adsorption properties. To confirm these results, SEM, FT-IR, XRF, XRD, Raman, and TGA techniques were employed. The fibrous carbon produced will expand the use of renewable carbon materials for removing various types of contaminants, including organic and inorganic pollutants in water, and numerous other industrial applications.

## Highlights

• Optimization of the production process of activated carbon from olive pomace.
• Activated carbon production requires soft and low-cost experimental conditions.
• Olive pomace activated carbon is characterized by a distinctive fibrous structure.
• The olive pomace-derived activated carbon fibers exhibit high adsorption capacities.

## Graphical abstract

**Figure:**
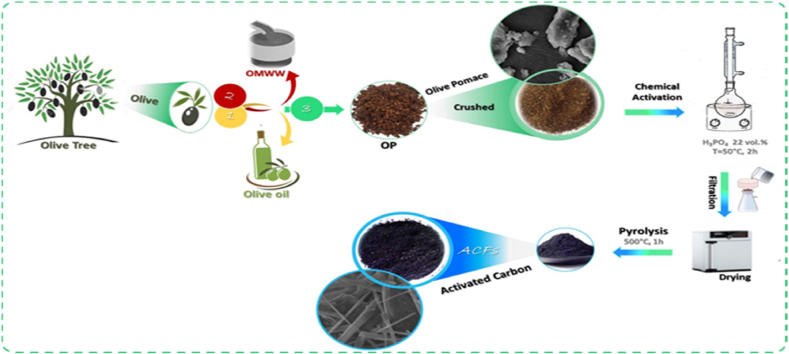

## 1 Introduction

In recent decades, global olive oil production has experienced significant growth, with the Mediterranean region emerging as a major consumer, thereby establishing the olive oil industry as a key agri-food sector [1]. However, this growth comes with environmental challenges, as the extraction operations involved in olive oil production generate substantial quantities of by-products, including olive pomace (OP) and olive mill effluent [1,2]. Approximately 80 % of the olive fruit remains unused after producing extra-virgin olive oil, resulting in OP, a viscous residue composed of water, residual skin, pulp, and stone fragments [3]. OP can pose serious environmental hazards if left untreated due to its high organic content [[3], [4], [5]].

Despite these challenges, OP presents a valuable opportunity for valorization, particularly through producing high-value products such as activated carbon (AC). The rich lignocellulosic composition of OP, comprising hemicellulose, cellulose, and lignin, makes it a suitable candidate for thermal decomposition processes that yield biochar precursors and subsequently activated carbon [6]. Extensive research has focused on utilizing OP as a sustainable precursor for activated carbon production, given the material's versatility in various industrial applications such as water purification, gas adsorption, and catalytic support [7,8].

At the same time, the effectiveness of activated carbon (AC) depends on its structural properties, notably its morphology, which can take the following forms: powdered (PAC), granular (GAC), spherical (SAC), and fibrous (ACF), each with distinct structural properties that influence its performance in specific applications. Synthesis conditions, including the choice of precursor, activating agent, and heat treatment parameters, play a crucial role in determining the final morphology of activated carbon [7,9,10]. Powdered activated carbon is generally obtained by mechanical grinding of carbonized precursors or by chemical activation, resulting in fine, high-surface-area particles suitable for rapid adsorption processes. Granular activated carbon, on the other hand, is produced by physical or chemical activation followed by sieving to obtain the desired particle size, making it ideal for fixed-bed adsorption systems. Spherical activated carbon is synthesized through processes such as emulsion polymerization followed by pyrolysis, resulting in uniform spheres with advantageous fluid dynamic properties in fluidized bed reactors. Finally, activated carbon fibers (ACFs), also known as carbon nanofibers or microfibers, are produced using electrospinning or chemical vapor deposition techniques. These fibers feature high surface area and microporosity, making them particularly effective for high-performance filtration applications [[16], [17], [18], [19]]. Recent studies have highlighted the potential of activated carbon with a fibrous structure (ACF), which has superior adsorption capacities due to its specific physical and chemical characteristics [11]. The production of carbon fibers is generally based on three distinct compounds and their respective ratios: cellulose, hemicellulose, and lignin derived from the raw material used in the process [[12], [13], [14], [15], [16], [17], [18]]. At the same time, OP is emerging as a promising renewable precursor for the preparation of carbon fibers due to its distinctive chemical composition.

Given OP's unique chemical composition, it appears to be a promising renewable precursor material for the preparation of various forms of activated carbon, including fibrous structures. However, obtaining the desired fibrous morphology from OP involves overcoming certain challenges associated with the nature of the preparation methods. In contrast, chemical activation using agents such as H_3_PO_4_, KOH, or H_2_SO_4_ offers a simpler, less energy-intensive alternative [6,[22], [23], [24], [25], [26]]. Among these agents, H_3_PO_4_ activation is particularly advantageous due to its ability to generate highly porous carbons under relatively mild conditions, producing large particle sizes suitable for applications such as water treatment [10].

This study aims to explore a simplified and efficient approach for producing porous ACFs using chemical activation with phosphoric acid, followed by low-temperature pyrolysis under air. The study examines the influence of key parameters on the synthesis of carbonaceous materials and their intrinsic characteristics, including the concentration of activating agents, the activation process, the pyrolysis temperature, and the duration of pyrolysis. The results should demonstrate the efficacy of the process in producing activated carbons with exceptional properties, particularly at lower temperatures, without the necessity for complex catalysts. This cost-effective and energy-efficient method represents a significant advancement in the sustainable synthesis of activated carbons from OP biomass, with potential applications in water treatment and industrial waste management.

## 2 Materials and methods

### 2.1 Collection, pre-treatment, and characterization of raw material

Olive pomace was collected during the 2020/2021 olive harvesting season from a modern industrial facility in the Taza region in Northcentral Morocco. This facility employs a three-phase centrifugal process to crush olives. Subsequently, the OP underwent a preliminary washing stage to eliminate dust and adherent impurities. The samples were then dried in an oven at 105 °C for 24 h. Then, the OP was finely crushed and sieved to isolate particles ranging between 0.25 and 0.5 mm. The constituents and composition of the OP are detailed in Table 1.

**Table 1:** Proximate analysis and chemical composition of the raw OP.

Proximate analysis of OP (%)		Chemical composition of OP (wt.%)	
***Moisture***	4.18	***Cellulose***	27.87
***Ash***	1.40	***Hemicelluloses***	25.50
***Volatile matter***	76.16	***Lignin***	30.02
***Fixed carbon***	23.84	***Extractives***	16.00
***Density (g/cm***^***3***^***)***	1.429		

### 2.2 Preparation of activated carbon ACFs

The process of producing activated carbon nanofibers from olive pomace involves two main steps, as shown in Fig. S1. In the first step, chemical activation of ACFs from OP is carried out. This involves impregnating 2.5 g of OP with a 100 ml solution containing 22 vol% phosphoric acid (H_3_PO_4_). The impregnation process occurs at 50 °C for 2h under magnetic stirring. Following filtration, the activated OP, now in paste form, undergoes washing with distilled water to remove excess H_3_PO_4_, followed by neutralization with 0.1 mol/L sodium hydroxide (NaOH). Subsequently, the material is dried in an oven at 50 °C for 24 h. In the second step, the activated OP is subjected to thermal treatment in a programmable muffle furnace in an inert atmosphere. The temperature is ramped up from room temperature to 500 °C at a rate of 5 °C/min. Upon reaching 500 °C, they are maintained at an isothermal stage for 60 min. The resulting carbonaceous product is stored in a desiccator until ready for use [27]. We studied the effects of several parameters, such as activating agent, pyrolysis time (ranging from 30 min to 4h), and pyrolysis temperature (ranging from 300 °C to 700 °C), to optimize the OP production process. Each experiment was repeated three times to ensure reproducibility and accuracy of results.

### 2.3 Activation and pyrolysis conditions with H_3_PO_4_

The activation of the OP was achieved by mixing the raw material, which had been washed, with different concentrations of H_3_PO_4_ solutions (2.5, 5, 10, 22, 42, 64 and 85 %) at 50 °C, which were kept constant for 2 h under magnetic agitation. Subsequently, the mixture was neutralization with sodium hydroxide 0.1 mol/L, is then dehydrated in an oven for one night at 105 °C. Then, the pyrolysis step was carried out in a muffle oven at different temperatures (300, 400, 500, 600, 700 °C at a rate of 5 °C/min) and times (0.5, 1, 2, 3, 4h) respectively in an air reactor (Fig. S1). The resulting carbonaceous product was cooled to room temperature and stored in a desiccator until ready for use.

### 2.4 Characterization of activated carbon ACFs

The aim of this study was to evaluate the quality and efficiency of ACFs by assessing three key parameters: activation yield, pyrolysis yield, and iodine number.

#### 2.4.1 Activation and pyrolysis yield

The mass yield represents an important quantitative measure of the process performance in the ACF preparation, indicating biomass mass loss during activated carbon elaboration. It is calculated as the ratio of final mass (m_f_) and initial mass (m_i_).

(1) $$Y\;\left(\%\right)=\frac{m_{f}}{m_{i}}*100$$

#### 2.4.2 Iodine number

The iodine number indicates activated carbon porosity, representing the quantity of iodine adsorbed by 1 g of carbon [28]. This was determined following ASTM designation D460786, titrating with sodium thiosulfate. The iodine solution concentration was calculated from the total sodium thiosulfate volume and dilution factor [9].

(2) $$\text{ION}=\frac{25.4*\left(20-\text{Vthio}\right)}{m_{\text{ACFs}}}$$

Where V_thio_ is the volume of Na_2_S_2_O_3_ solution (mL), m_ACFs_ is the masse of activated carbon (mg).

#### 2.4.3 Physico-chemical and structural characterizations

Physicochemical characteristics of OP and ACFs were determined for moisture, ash content (ASTM D3174-04), and volatile matter (ASTM D3175-89a), alongside lignocellulosic composition analysis including hemicellulose, cellulose, and lignin content [29]. Actual powder density was determined using a Micromeritics Accupyc II 1340 gas pycnometer. The zero-charge point pH (pH_PZC_) was determined using Lopez Ramon's method [30]. Specific surface area (BET) was measured by nitrogen adsorption at 77 K (Nova 4220e, Quantachrome Instruments) following the Brunauer Emmett and Teller (BET) method [31]. X-ray fluorescence spectrometry (XRF) (Epsilon 5, PANalytical) assessed metallic element presence. Morphological analysis was conducted using scanning electron microscopy (SEM) JEOL-IT500HR with EDS analyzer. Surface functional groups were determined via Fourier transform infrared spectroscopy FT-IR spectra were recorded on a PerkinElmer 1720 FT-IR spectrometer in the 600-4000 cm^−1^ wavenumber range, with 10 scans performed at 1 cm^−1^ resolution. X-ray diffraction (XRD) analysis was performed on a powder diffractometer (XRD, PANalytical X'Pert-Pro), and Raman spectra were obtained using a Laser Resonant Raman-transient ﬂuorescence spectrometer (Lab RAM HR Evolution, Horiba) using a 532 nm laser. Thermogravimetric analysis (TGA) measured mass variation with temperature on an SDT Q600 thermo-balance (TA Instruments) under a controlled atmosphere ramping from room temperature to 700 °C at 10 °C/min.

## 3 Results and discussion

### 3.1 Optimal conditions for ACFs production

#### 3.1.1 Effect of the activating agent H_3_PO_4_ on activation yield

**Fig. 1:**
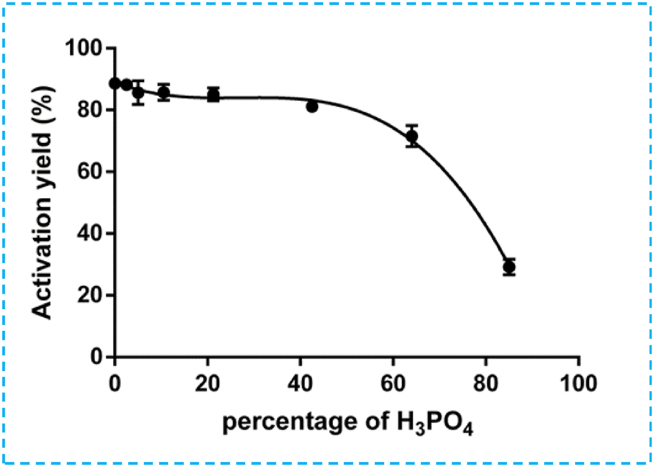
Effect of percentage of activating agent H_3_PO_4_ on activation yield at 50 °C for 2h.

The impact of using phosphoric acid as the activating agent on the activation yield of olive pomace was investigated. OP was impregnated with different percentages of phosphoric acid and subjected to steady magnetic stirring at 50 °C for 2 h. The results indicate a slight decrease (approximately 10–15 %) in yield (repeated experiment) initially, followed by a relatively stable yield with and without H_3_PO_4_ (ranging from 2.5 to 42.5 %) (Fig. 1). This initial mass loss can be attributed to the solubility to the solubility of the raw material in the water solvent, and the physicochemical effects of the H_3_PO_4_ precursor on the lignocellulosic and extractive components present in the raw OP. Additionally, losses during filtration and washing operations may contribute to this mass loss [31,32]. However, a significant drop is observed from 81 to 30 % as the H_3_PO_4_ percentage increases from 42.5 to 85 %. This decrease suggests the decomposition of organic raw material into volatile substances due to the reaction between high concentrations of H_3_PO_4_ and the lignocellulosic compounds present in OP. 10.13039/100014337Furthermore, Supporting Information (Figs. S4 and S5) shows a positive correlation between experimental values and those predicted by the model for all responses to preparation conditions. Based on these results, the optimal conditions for maximum yield are identified as follows: (i) an activation temperature of 50 °C, (ii) an activation time of 2 h, and (iii) an activating agent concentration of approximately 22 % H_3_PO_4_.

#### 3.1.2 Effect of pyrolysis temperature on pyrolysis yield

**Fig. 2:**
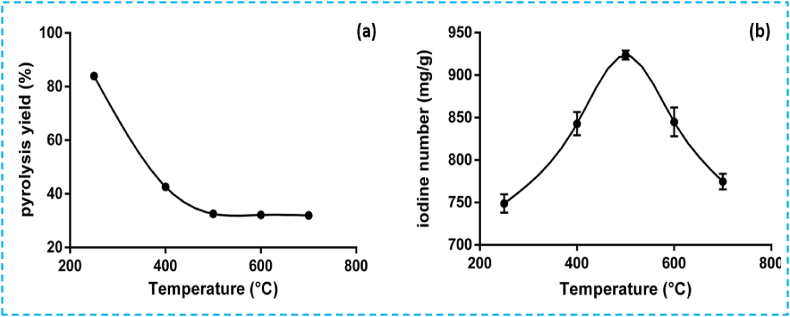
Effect of pyrolysis temperature on (a) pyrolysis yield and (b) iodine value.

To evaluate the influence of pyrolysis temperature on yield, temperatures ranging from 250 °C to 700 °C were investigated. The results are consistent with established thermochemical phenomena, indicating that elevated temperatures lead to increased degradation of biomass macromolecules, resulting in the formation of organic and inorganic matter. Consequently, mass yields exhibit a notable decrease as pyrolysis temperature rises, with a consistent plateau observed beyond 500 °C (illustrated by a 43 % decrease, as shown in Fig. 2a). Similar trends have been reported in the literature. Indeed, the reduction of the activated carbon yield was associated with the increase in pyrolysis temperature during phosphoric acid activation [33]. Accordingly, an increase in the carbonization temperature has been associated with a proportional decrease in the mass yield for activated carbon derived from date stones [34]. Similar results were obtained by Wu FC et al. [35], where highly porous carbon was prepared from Fir wood by KOH etching and CO_2_ gasification. Regarding the iodine value, a dual behavior was observed. A continuous increase of this parameter was observed up to 500 °C, reaching its peak at 923 mg/g. Subsequently, a decrease in both iodine value and yield was observed at higher pyrolysis temperatures (as depicted in Fig. 2b). Pyrolysis at 600–700 °C can induce shrinkage of the carbon structure, resulting in decreased adsorption capacity [9]. This phenomenon arises from the decomposition of oxygen groups at higher temperatures, adversely affecting porosity development and the adsorption properties of ACFs [20,36]. To investigate the effect of pyrolysis parameters (residence time and pyrolysis temperature), the percentage of the activating agent was selected according to the obtained iodine value (Fig. 2b). Therefore, the H_3_PO_4_ percentage was fixed at 22 %, which corresponds to the higher iodine value recorded (923 mg/g ± 5, as shown in Fig. 2b).

#### 3.1.3 Effect of pyrolysis time on pyrolysis yield

**Fig. 3:**
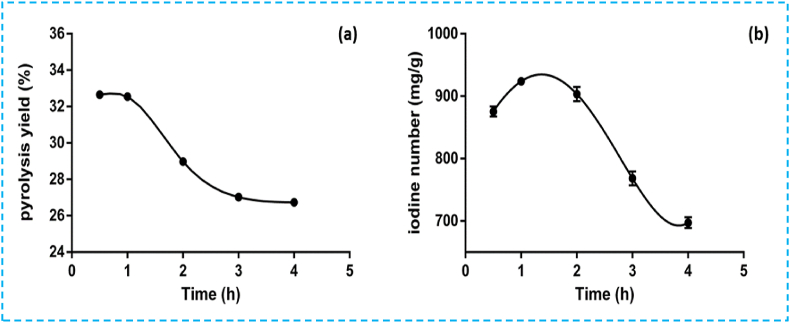
Effect of pyrolysis time on (a) pyrolysis yield and (b) iodine value.

To investigate the impact of residence time, the parameter was varied from 30 min to 4 h at a constant pyrolysis temperature of 500 °C, while keeping other parameters of the chemical activation step constant. The pyrolysis yield and iodine number were then monitored as a function of residence time (Fig. 3a). Therefore, optimal pyrolysis yield and iodine value were observed at 1 h. Beyond this duration, a slight decrease in pyrolysis yield and iodine number was noted (Fig. 3b). This decrease stemmed from the development of macropores due to micropore coalescence or enlargement and pore burning at higher reaction times [9]. The same results regarding iodine values as a function of pyrolysis time were observed by other researchers [37,38].

#### 3.1.4 Effect of the activating agent H_3_PO_4_ on pyrolysis yield

**Fig. 4:**
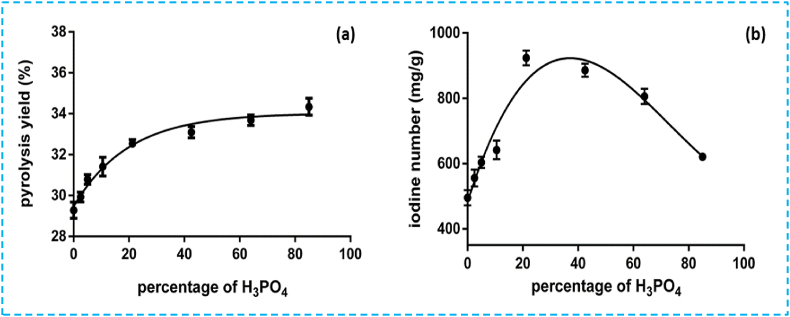
Effect of the percentage of activating agent on (a) pyrolysis yield and (b) iodine value.

To explore the chemical activation influence on the pyrolysis yield, pyrolysis was conducted for 1 h at 500 °C while varying H_3_PO_4_ percentages, as shown in Fig. 4a. The results revealed that as the percentage of phosphoric acid increased, there was a corresponding increase in pyrolysis yield. Additionally, higher percentages of H_3_PO_4_ led to a notable improvement in iodine number (Fig. 4b), indicating the development of micropores and enhancing the adsorption capacity of the resulting activated carbons. However, when the H_3_PO_4_ percentage exceeded 22 %, a decreased iodine number was observed (Fig. 4b), potentially related to the enlargement or destruction of the developed micropores caused by the high oxidizing nature of H_3_PO_4_. This decrease could significantly reduce the adsorption capacity of the activated carbon [9]. In summary, varying the activating agent percentage resulted in activated carbon yield ranging from 27.10 to 64.57 %, with corresponding variations in both pyrolysis yield and iodine numbers (ranging from 27.10 to 64.57 %, and from 293 mg/g to 923 mg/g, respectively). Furthermore, additional research conducted by other authors is employed to assess the effect of the activating agent by examining the degree of impregnation ratio (IR). This allows the determination of the amount of phosphorus used for impregnation based on the weight ratio of impregnation (IR) according to the relationship described in equation (3) [32,39].

(3) $$\text{IR}=\frac{m_{p}}{m_{\text{op}}}*100$$

where, m_p_ represents the mass of phosphoric acid (in grams), which is determined from the density of H_3_PO_4_, and m_op_ is the mass of OP (in grams).

In order to achieve the optimal conditions, with an H_3_PO_4_ percentage approaching 22 % by volume, it was determined that an IR of approximately 1.26 was necessary. These findings align with those of previous studies, which demonstrate the impact of the activating agent percentage on the iodine number of carbonaceous adsorbents with significant surface area and surface model [40].

### 3.2 Characterization of activated carbon ACFs

A comprehensive understanding of the physicochemical and structural properties of any given material is essential for elucidating a multitude of phenomena, including adsorption and desorption. This section presents the principal characteristics of activated carbon derived from OP, which we have designated ACFs. The characterization presented concerns the ACF sample obtained under the optimum synthesis conditions, which were identified as a phosphoric acid concentration of 22 % (vol%), chemical activation for 2 h at 50 °C, followed by 1-h pyrolysis at 500 °C under an inert atmosphere.

#### 3.2.1 Proximate and chemical composition analysis

The valorization of biomass through thermochemical transformation depends on its nature and properties. The proximate analysis enables the determination and evaluation of the properties of OP and the activated carbon fibers (ACFs) derived from it via chemical activation with H_3_PO_4_ and subsequent pyrolysis. The results of the proximate analysis are tabulated in Table 1, Table 2. The biomass exhibits high volatile content and low ash content, rendering OP a suitable precursor for conversion to activated carbon. Similar trends have been reported in previous studies, such as the carbonization of paulownia wood with phosphoric acid by Yorgun et al. [2] and the preparation of activated carbon from cherry stones by physical activation by Olivares-Marín et al. [41]. As expected for activated carbon, low-temperature pyrolysis induces a significant increase in ﬁxed carbon content and a decrease in volatile content, while the ash content escalates approximately threefold for ACFs compared to OP.

**Table 2:** Results of EDS and chemical composition analysis of ACFs.

Elemental analysis EDS (wt %)		Chemical composition (%)	
***C%***	84.08	***Moisture***	1.71
***O%***	10.34	***Ash***	4.15
***N%***	0.41	***Volatile matter***	12.23
***S%***	0.28	***Fixed carbon***	87.77
***P%***	1.46	***Density (g/cm***^***3***^***)***	1.595
***Na%***	1.85	***Porosity (%)***	36.55
***Al***	0.56	***Iodine number (mg/g)***	923
***Si***	1.02	***SBET (m***^***2***^***·g***^***−1***^***)***	1400

With regard to the chemical composition analysis (Table 1), lignin represents the predominant component, constituting 30 wt% of the total composition, followed by cellulose at 27.87 % wt.%. Hemicellulose constitutes 25 % wt.% of the biomass, with an additional fraction comprising organic extractives, representing 16 wt%. These findings are consistent with those reported in other studies [19].

#### 3.2.2 Point of zero charges pHpzc

**Fig. 5:**
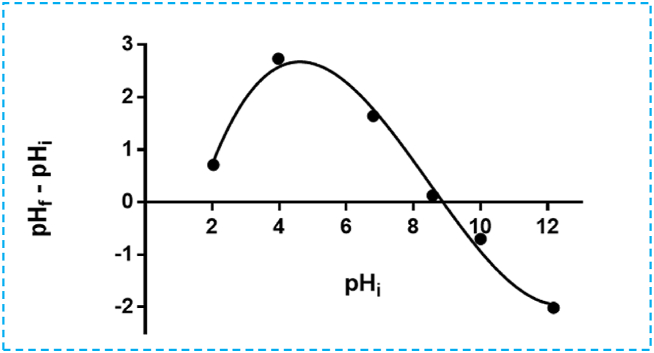
Point of zero charge titration (pHpzc) of the ACFs.

The pHpzc value serves as a defining characteristic that indicates the pH at which the surface charge of the adsorbent becomes neutral. As illustrated in Fig. 5, the pHpzc of the elaborated activated carbon (ACFs) is determined. When the pH exceeds the pHpzc, the material surface carries an overall negative charge, facilitating the adsorption of cations. On the other hand, for pH below pzc, anion adsorption will be preferred. Conversely, when the pH is below the pHpzc, anion adsorption becomes more favorable. Based on the findings, the activated carbon exhibits a basic pHpzc of 8.8. Below this pH, the surface becomes positively charged, while above it, the surface becomes anionic. These results are in agreement with the research conducted by Joachim Krou [42], who observed that the zero-charge point of various coals elaborated from deficient biomass falls within the basic pH range of 6.2–9.7. Also, other studies have reported acidic zero charge points for coal, such as those by Liu et al. and Lopez-Ramon et al. [30,43].

#### 3.2.3 Textural characterization by SEM and EDX

**Fig. 6:**
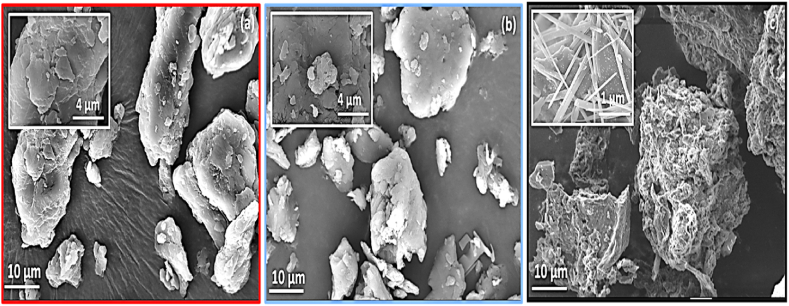
SEM images: (a) raw OP-R, (b) OP-A after activation, (c) activated carbon ACFs.

Textural characterization through SEM and EDX analysis was conducted to examine the external morphology of raw OP, activated OP, and activated carbon, allowing for a comparative assessment of the effects of activation via phosphoric acid and pyrolysis. The SEM micrographs depicting the three adsorbents are provided in Fig. 6. As illustrated in Fig. 6a, the surface of the unprocessed OP exhibited a curled shape, a compact appearance, and an absence of pores or cavities. This morphology is consistent with the findings of previous research by M.S. Shamsuddin et al. [44], which noted similar characteristics in kenaf core fiber (KF) due to the presence of cellulose, hemicelluloses, and lignin in the raw material, resulting in a low or negligible BET surface area. However, activation by H_3_PO_4_ resulted in a roughened surface and the formation of irregular pores in the activated OP, as shown in Fig. 6b. Notably, the SEM analysis of activated carbon (Fig. 6c) revealed highly developed textural and structural properties, which were characterized by a porous and fibrous morphology (Fig. S2). The carbon material presents a highly porous morphology with a range of pore sizes, along with a fibrous structure comprising lengths and diameters of approximately 2–3 μm and 100–200 nm, respectively. Moreover, the SEM images revealed the existence of cavities on the external surfaces of the activated carbon, indicating the development of a substantial specific surface area by the supports. These results were confirmed by the remarkable values of the iodine index (exceeding 923 mg/g) and specific surface area measurements (exceeding 1400 m^2^/g) of the tested supports. Furthermore, the activation and pyrolysis reactions were observed to create pores of varying sizes, predominantly macros and mesopores. This finding was corroborated by the research of Thanapal et al. [45], who observed an increase in surface porosity of char after the charring process, resulting in a large surface area with small voids on the surface. Additionally, the formation of these voids contributed to the decomposition of volatile materials in the biomass. Activation at 500 °C with H_3_PO_4_ resulted in the creation of more pores, and substantial removal of volatiles. It is postulated that the combination of air and low-temperature treatment facilitates the development of phosphate and polyphosphate bridges, which play a role in connecting crosslinking lignin fragments [31,46].

**Fig. 7:**
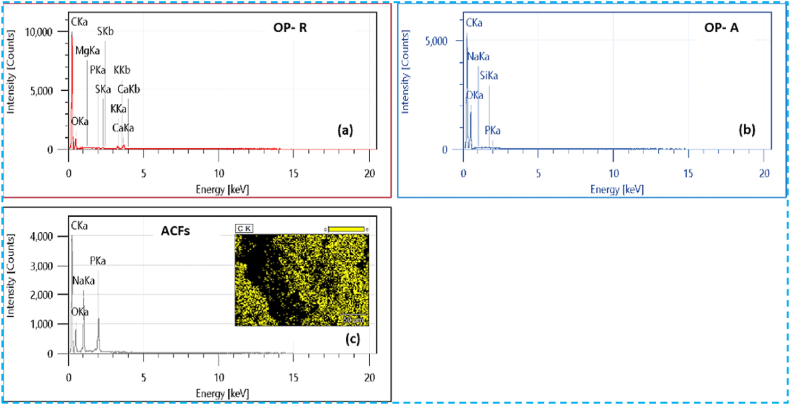
EDX spectra of raw OP-R (a), activated OP-A (b), and activated carbon ACFs (c).

SEM-linked energy dispersive X-ray (EDX) analysis was utilized to assay the mineral species existing on the surface of the three samples. The EDX spectra related to the crude OP, activated OP, and the produced activated carbon are presented in spectra a, b, and c of Fig. 7. The findings indicate that both the crude and activated pomace primarily comprise carbon, constituting 75.01 % and 76 %, respectively, in addition to traces of mineral matter. This composition renders the material an ideal precursor for the preparation of activated carbon. Upon pyrolysis, the EDX spectrum of activated carbon (Fig. 7c) illustrates an increase in carbon content on the carbon surface reaching 84.08 % (Table 2), along with the presence of a phosphorus peak. This observation aligns with the use of phosphoric acid as the activating agent. It is noteworthy that, the persistence of phosphoric acid residues on the activated carbon surface post-washing suggests incomplete removal, which may result in their potentially leading to its incorporation into the material matrix [47]. These findings are in accordance with the research conducted by Benadjemia et al. [48], which demonstrated a correlation between impregnation rate, increased carbon content, and decreased oxygen content.

#### 3.2.4 FT-IR Fourier - infrared spectroscopy analysis

**Fig. 8:**
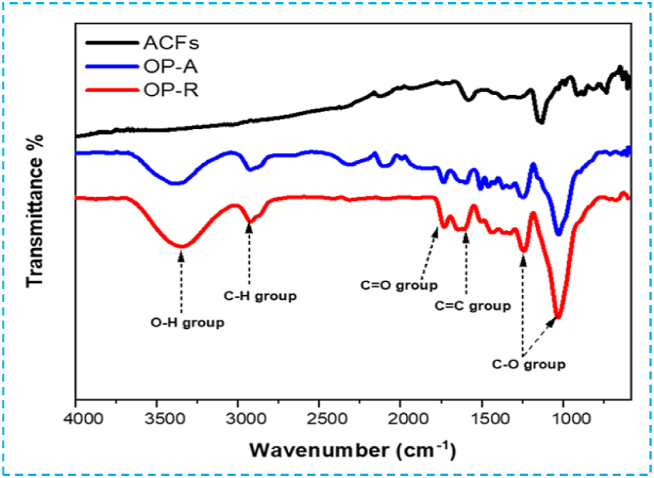
FT**-**IR spectra of raw OP-R (red), activated OP-A (blue) and activated carbon ACFs (black).

It is generally accepted that the development of pore structure is influenced by many factors, including inorganic impurities and internal carbon structure [9]. The FT-IR spectra presented in Fig. 8 illustrate the development of surface textures in raw OP, activated OP, and prepared activated carbon. According to literature, it has been observed that both raw OP and activated pomace exhibit an IR band around 3400 cm^−1^, attributed to the vibrational stretching of -OH hydroxyl groups stemming from lignin, cellulose, and hemicellulose in the raw materials, which contain carboxyl and alcohols [49,50]. This band completely disappears in the spectrum of activated carbon, replaced by a carbon structure. This trend suggests that the -OH groups on the surface of OP and OP-A have been reduced and disappear after carbonization. Moreover, both OP and activated pomace display a distinct absorption peak at 2920 cm^−1^, representing the aliphatic vibration of C-H. However, the intensity of this peak in activated carbon gradually decreases, nearly disappearing. These results indicate a gradual decomposition of cellulose following carbonization, which promotes the formation of a porous carbon structure [49].

The spectra show a pronounced band at 1630 cm^−1^, which can be attributed to the stretching vibration of the C=O bonds in lactone and carbonyl groups [37], as well as to the C=C groups conjugated to the aromatic rings in the three samples [26]. Additionally, the band observed at 1000-1300 cm^−1^ is typically associated with oxidized carbons and has been assigned to C-O stretching in acids, alcohols, phenols, ethers, and/or ester groups [50]. Nevertheless, this band also reflects the presence of phosphorus and phosphocarbon compounds in H_3_PO_4_ activated carbon. The FT-IR spectroscopy results indicate that the produced activated carbon is abundant in surface functional groups [51].

#### 3.2.5 X-ray diffraction (XRD) and (XRF) analysis

**Fig. 9:**
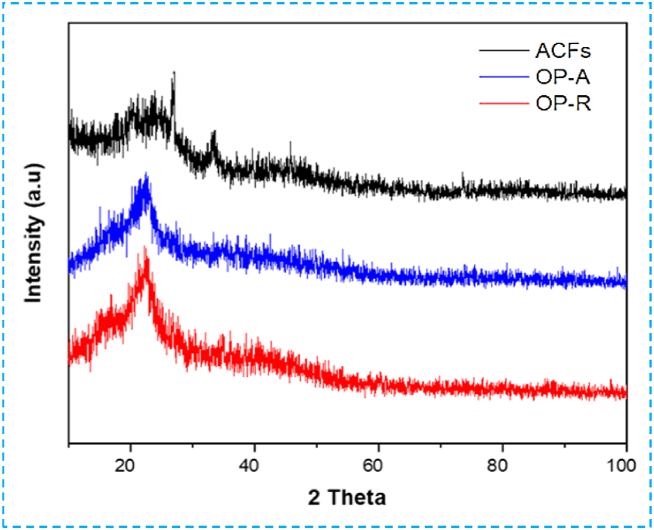
XRD spectra of OP-R (red), activated OP-A (blue), and activated carbon ACFs (black).

X-ray diffraction (XRD) and X-ray fluorescence (XRF) analyses were conducted to assess the structural and molecular composition of the samples. The XRD diffractogram of the OP, activated OP, and prepared activated carbon aggregates reveals an amorphous structure for all three samples, as shown in Fig. 9. Notably, activated carbon shows additional peaks likely induced by phosphoric acid used during chemical activation. Furthermore, the diffractogram indicates the presence of a peak at approximately 2θ at 22–26°, suggesting small, irregular domains of graphene sheets stacking [52], and a weak peak around 43°, indicating the presence of honeycomb structures formed by sp^2^ hybridized carbons [53]. These characteristics are typical of amorphous carbon, as supported by previous studies [49,54].

XRF analysis was carried out to identify the molecular composition of the activated carbon derived from OP. Results listed in Table 3 reveal the molecular composition of OP-R raw material, showing a prominent presence of calcium (CaO) and potassium (K_2_O) as the main metallic species in the biomass [55]. It is important to note that these elements progressively decrease upon contact with phosphoric acid and during carbonization, while the percentage of phosphorus (P_2_O_5_) increases thanks to chemical activation by the acid. Moreover, phosphorus remains present in the activated carbon structure obtained after pyrolysis of activated carbon derived from pomace.

**Table 3:** XRF analysis results of OP-R, OP-A and ACFs (Bal = balance).

XRF (wt. %)										
**components**	Bal	SiO_2_	TiO_2_	Al_2_O_3_	Fe_2_O_3_	CaO	K_2_O	**P**_**2**_**O**_**5**_	MnO	MgO
**sample**										
***OP- R***	95.19	0.46	0.01	–	0.13	1.97	2.05	**0.19**	–	–
***OP-A***	93.92	0.51	0.01	–	0.03	0.06	0.02	**5.45**	–	0.38
***ACFs***	90.38	1.16	0.02	0.10	0.15	0.29	0.10	**7.58**	–	0.37

Bal = balance.

#### 3.2.6 Raman spectroscopy

**Fig. 10:**
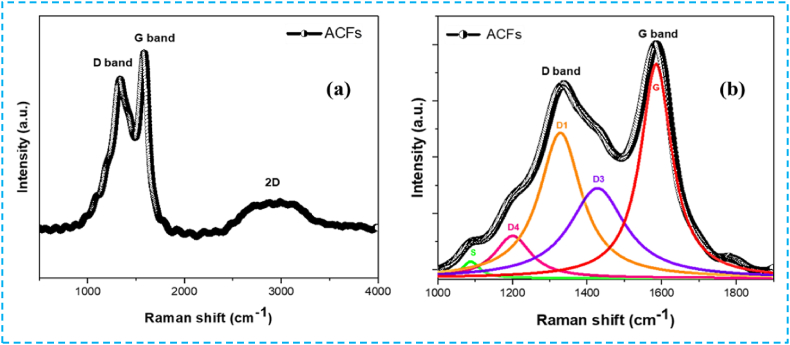
Raman spectra of ACFs (a) and Curve-fitting of Raman spectra (b).

The Raman spectrum of ACFs is depicted in Fig. 10. Activated carbon exhibits two distinct intrinsic peaks, approximately at 1335 cm^−1^ and 1580 cm^−1^, corresponding to the D and G bands, respectively. The D peak signifies the presence of a disordered and defective carbon structure [54,56], while the G band represents the in-plane vibration of the sp^2^ graphitic carbon structure [57]. Furthermore, the spectrum displays an additional band at a higher wavenumber, ranging from 2600 to 3450 cm^−1^ (2D), indicating the presence of carbon harmonics and revealing the graphitic nature of the activated carbon material, which is few-layered [58]. According to the curve fitting displayed in Fig. 10b, the sample's Raman spectra's deconvolution normally produces five Gaussian distributions: The G, D1, D3, D4, and S bands. Thus, the ratio of the integrated areas of the D-band peaks to those of the G-band (AD/AG) is 0.83, indicating a high degree of carbon disorder in ACFs.

#### 3.2.7 Thermogravimetric analysis TGA

**Fig. 11:**
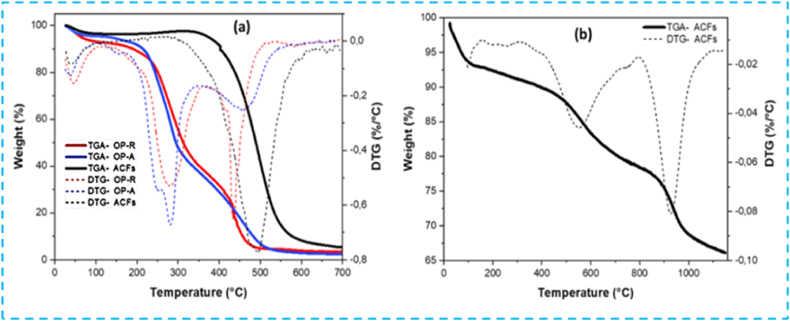
TGA analysis in controlled atmosphere (a) TGA/DTG of OP-R (red), activated OP-A (blue), and activated carbon ACFs (black). (b) TGA/DTG under N_2_ atmosphere of activated carbon ACFs.

Thermogravimetric analysis (TGA) provides insights into the thermal behavior of the precursor materials. Fig. 11 illustrates the TGA-derived thermogravimetric and DTG-thermogravimetric profiles for OP, activated olive pomace (OP-A), and activated carbon (ACFs). The curves reveal three distinct weight losses in the thermograms of raw OP and chemically activated OP-A. The initial weight loss, occurring from room temperature to 105 °C, corresponds to rapid moisture loss, constituting approximately 7 % of the sample weight. The second weight loss, accounting for 56 %, is attributed to the degradation onset of cellulosic and hemicellulose components. This phase is characterized by hemicellulose degradation predominating between 240 °C and 300 °C, followed by cellulose degradation approximately between 320 °C and 400 °C. The DTG curve gives a better indication of the thermal decomposition of hemicelluloses and cellulose, marked by a prominent asymmetrical peak. The third mass loss, comprising 26 % within the temperature range of 380–550 °C, corresponds to lignin thermal decomposition, which initiates earlier and exhibits greater thermal stability to the other constituents [59]. Furthermore, the DTG curve reveals a more intense peak for OP-R observed at temperatures between 400 and 520 °C, with reduced intensity for OP-A due to phosphoric acid utilization. Mass loss stabilizes around 480 and 560 °C for OP and activated OP, respectively (Fig. 11a).

The TGA curve of ACFs indicates a two-stages decomposition process. The initial stage, occurring below 100 °C, involves approximately 4 % mass loss attributed to small molecules desorption and water evaporation, derived from sample moisture and reactions with H_3_PO_4_ at low temperatures [60]. Subsequently, ACFs demonstrate thermal stability up to 400 °C, with the release of light-volatile compounds originating from cellulose and hemicellulose degradation [9]. The formation of micropores and mesopores is facilitated by phosphoric acid's reaction with biopolymer (hemicellulose and cellulose) to form cross-linked biopolymer fragments connected by phosphate and polyphosphate bridges [48]. The second decomposition stage observed between 450 and 600 °C, is associated with sample carbon combustion, with a more pronounced peak evident in the DTG curve (Fig. 11a). Furthermore, the incorporation of phosphorus groups and lignin significantly improved carbon nanofiber thermal stability [61,62]. In addition, the TGA control curve under nitrogen for ACFs presented in Fig. 11b, reveals three degradation stages, with total carbon weight loss ranging from 31 to 33 %. These stages include moisture evolution, volatile substance release at 480–620 °C, and carbon matrix carbonization between 900 and 1000 °C. Notably, the thermal stability of our material is similar to that of carbon nanofibers obtained through alternative methods [61,62].

Table 4 provides a summary of several the conditions for preparing of the activated carbons prepared from various byproducts referenced in the literature. As can be observed, a number of previous studies have used a highly concentrated activation agent [[10], [11], [12], [13],^21^,^22^,^34^,^63^], long pyrolysis times [38,52], and the high pyrolysis temperatures (>600 °C) [20,21,32,38,52] which is expected to make the manufacturing process more expensive and relatively complex. In comparison, our process differs from other processes in that it utilizes a dilute concentration of activating agents (H_3_PO_4;_ 22 vol%), and a low-temperature pyrolysis condition without inert gas (500 °C for 1 h). By using this approach, our process becomes simpler, more economical, and more environmentally friendly. Therefore, the ACFs obtained have both a carbon fiber morphology and a very high surface area (1400 m^2^/g), which are comparable or greater than the work listed in Table 4.

**Table 4:** Comparing the conditions for preparing AC with the different by-products in the literature.

Precursor	Activating Agent	Pyrolysis conditions	*Morphology AC/Surface Area (m*^*2*^*/g)*	Ref
Olive pomace	ZnCl_2_/H_3_PO_4_ (85 %)	550 °C (30 mn), under N_2_	Uneven and rough texture/650.9	[10]
Cucumis melo	H_3_PO_4_ (98 %)	300–500 °C (1–3 h)	Uneven and rough texture/407.94	[11]
Rubber fruit shells	H_3_PO_4_ (85 %)	500 °C (1h)	Carbon nanofibers texture/63	[13]
Acacia leaves	0.5 M KOH	600 °C (2h), under N_2_+ CO_2_ (2.5h)	Carbon nanofibers texture/1281	[19]
Sour cherry stones	ZnCl_2_	900 °C (2h), under N_2_	Uneven and rough texture/1704	[20]
polyethylene terephthalate	H_2_SO_4_ (70 %)	800 °C (90 min), under N_2_	Uneven and rough texture/537	[21]
Coffee grounds	H_3_PO_4_ (85 %)	400–800 °C (1–3h), N_2_ + CO_2_	Folded and rough texture/720.9	[32]
Date palm stones	KOH/NaOH	Microwave irradiation/700 W (10 min) under N_2_	Folded and rough texture/715.30	[34]
Paper mill sludge	KOH	800 °C (3h) under N_2_	Irregular and rough texture/885	[38]
Rice husks	NaOH	500 °C (3h), 750 °C (90 min) under N_2_	Irregular and rough texture/2176	[52]
Leaves of sugar beet	H_3_PO_4_ (85 %)	550 °C (2h)	Spheroidal and rough texture/700.71	[63]
**Olive pomace**	**H**_**3**_**PO**_**4**_**(22 %)**	**500°C (1h)**	**Carbon fibers and rough texture/1400**	**This Study**

## 4 Conclusion

This study presents an efficient method for chemically activating olive pomace to produce porous activated carbon with a fibrous structure (ACFs) using H_3_PO_4_. By analyzing the effects of key parameters during the preparation process, we have identified a pathway to achieve high-quality activated carbon with enhanced efficiency and reduced costs. Through rigorous experimentation, the optimal conditions were identified: a phosphoric acid concentration of 22 % (vol%), chemical activation for 2 h at 50 °C, followed by 1 h pyrolysis at 500 °C low temperature under air. The resultant carbon material exhibits a distinctive structure, characterized by pronounced microporosity and a composition rich in nano and microfibers. The results were corroborated by comprehensive spectroscopic analyses, including scanning electron microscopy (SEM), Fourier transform infrared spectroscopy (FT-IR), X-ray fluorescence (XRF), X-ray diffraction (XRD), Raman spectroscopy, and thermogravimetric analysis (TGA). Once the process parameters were optimized, the activated carbon fibers demonstrated high adsorption capacities. It is noteworthy that an iodine value of 923 mg/g and a BET surface area of 1400 m^2^/g were recorded. These findings indicate the potential of nanofibrous activated carbon derived from OP as an effective solution for the removal of diverse pollutants, including organic and inorganic contaminants, from water sources and industrial discharges such as olive mill wastewater and tannery rejects. Moreover, this material displays potential as a cost-effective resource for future energy storage applications, including use as a supercapacitor or an anode for lithium-ion batteries.

## Ethical Approval

Not applicable.

## Funding

The work was financially supported by the VGOLIVES Project, the authors would like to thank the support from the Ministry of Energy, Mining, Water and Environment, Morocco. The findings achieved herein are solely the responsibility of the authors.

## Availability of data and materials

Datasets will be made available on request.

## CRediT authorship contribution statement

**Imad Alouiz:** Writing – review & editing, Writing – original draft, Validation, Software, Methodology, Investigation, Formal analysis. **Mouhssine Benhadj:** Writing – review & editing, Writing – original draft, Validation, Resources. **Elmontassir Dahmane:** Writing – review & editing, Writing – original draft, Validation. **Mohamed Sennoune:** Writing – review & editing, Writing – original draft, Validation. **Mohamed-Yassine Amarouch:** Writing – review & editing, Writing – original draft, Validation, Supervision. **Driss Mazouzi:** Writing – review & editing, Writing – original draft, Validation, Supervision, Investigation.

## Declaration of competing interest

The authors declare no competing interests.
