# Supplementary material for: Elaboration of fibrous structured activated carbon from olive pomace via chemical activation and low-temperature pyrolysis
Source: Heliyon. 2024 Oct 2;10(20):e38886. doi: 10.1016/j.heliyon.2024.e38886 (PMC11533574; doi:10.1016/j.heliyon.2024.e38886)
Supplement: Multimedia component 1 [file mmc1.docx]

**Supplemental information**

Elaboration of Fibrous Structure Activated Carbon from Olive Pomace via Chemical Activation and Low-Temperature Pyrolysis

Imad Alouiz, Mouhssine Benhadj, Dahmane Elmontassir, Mohamed Sennoune, Mohamed‑Yassine Amarouch, and Driss Mazouzi*

*R.N.E Laboratory, Multidisciplinary Faculty of Taza, University Sidi Mohamed Ben Abdellah, Fez, Morocco.*

*** Correspondance to:**

Prof. Driss Mazouzi, PhD

R.N.E Laboratory, Multidisciplinary Faculty of Taza,

University of Sidi Mohamed Ben Abdellah- Fez, Morocco.

Phone: +212535211976

Fax: +212535211978

E-mail: [driss.mazouzi@usmba.ac.ma](mailto:driss.mazouzi@usmba.ac.ma)

**Preparation of activated carbon-ACFs by chemical activation with H_3_PO_4_**

**Fig. S1:** Schematic representation of the process of producing activated carbon from olive pomace


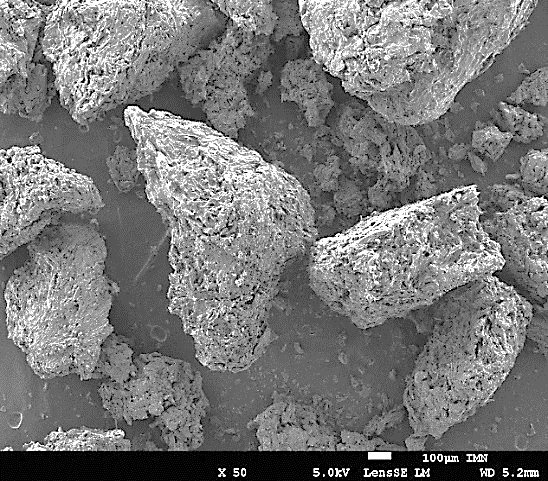

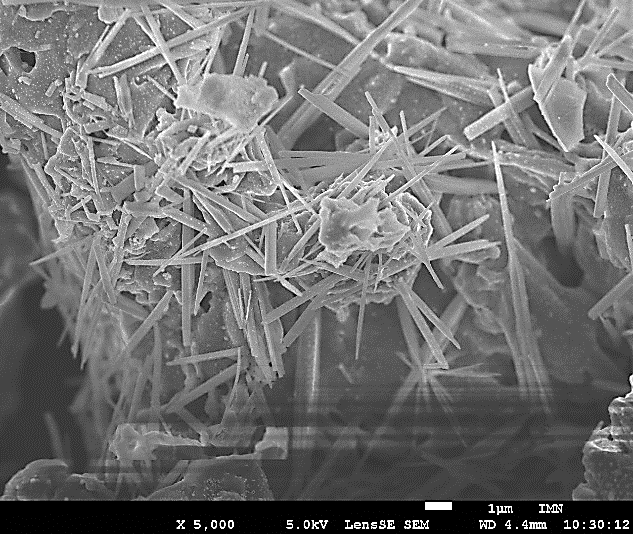

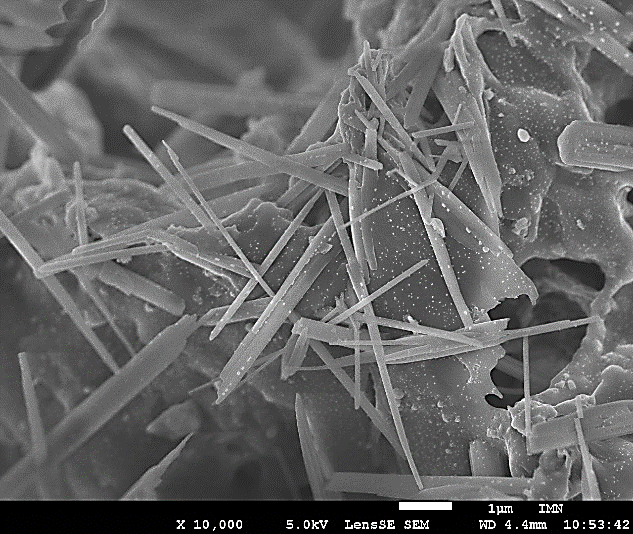

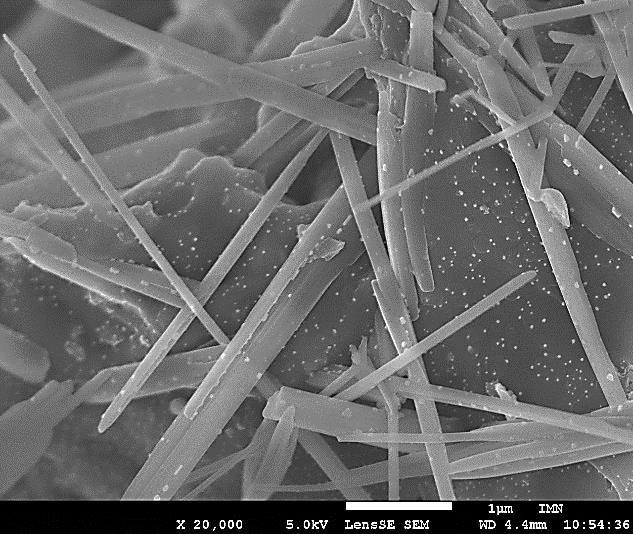


**Fig. S2**: SEM images of activated carbon (ACFs) at different magnifications. (a)-100µm, 50X; (b)-1µm, 5000X; (c)-1µm, 10000X; (c)-1µm, 20000X.

**Experimental design for the optimization of the preparation conditions of a highly efficient activated carbon derived by olive pomace using H_3_PO_4_ activation**

1. Design of experiment for carbonization of olive pomace

In order to optimize the texture and porosity of the ACFs produced, the steps for preparing this material through a chemical activation process have been optimized. In this sense, two analytical methods were chosen to monitor the ACFs production: (i) ***Method 1 used the influence of the principal factors*** by understanding the physicochemical mechanism underlying olive pomace chemical activation and pyrolysis, factors that are influencing these reactions were separately studied. Therefore, the investigated factors were: the phosphoric acid percentage, pyrolysis parameters (residence time and pyrolysis temperature). (ii) ***Method 2 used the experimental research methodology*** by optimizing the ACFs production in terms of activation and pyrolysis yields, the experimental research method was used [1-2]. In this sense, a factorial experimental design including three factors was developed: (A) the activating agent concentration, (B) activation temperature, and (C) activation time. Each of these factors takes two levels (Table S1).

**Table S1.** Experimental domain of factors

|  | Low levels (-1) | High levels (+1) |
| --- | --- | --- |
| A: Activating agent percentage (%) | 2.5 | 85 |
| B: Activation temperature (°C) | 50 | 100 |
| C: Activation time (h) | 2 | 24 |

The postulated mathematical model describing direct and interaction effects on the ACFs quality and quantity was defined by the following equation:

***Y = b_0_ + b_1_ A + b_2_ B + b_3_ C+ b_12_ A.B + b_13_ A.C + b_23_ B.C + b_123_ A.B.C*** (1)

Where Y is the response to be modeled; A, B and C are the studied factors; AB, AC, BC and ABC are interactions between factors; and b_i_ is the factors effects.

In the current work, the ACFs quality and efficiency were determined by measuring the activation yield, pyrolysis yield and iodine number

2. Experimental Research Methodology

- **Response analysis and interpretation**

To optimize the ACFs production in terms of activation and pyrolysis yields, the experimental research method was used. In this sense, a factorial experimental design including three factors was developed: (A) the activating agent concentration, (B) activation temperature, and (C) the activation duration.

The responses variables were activation yield (Y1) and pyrolysis yield (Y2). Each response was used to develop an empirical model that correlated the response to the activated carbon preparation variables (equation 1 and 2). The complete design matrix and the responses variables are given in Table S2.

**Y_1_** = 99.26 – 0.7958 A – 0.1762 B – 0.6553 C + 0.001538 A.B + 0.007587 A.C + 0.006407 B.C – 0.000090 A.B.C (1)

**Y_2_** = 28.69 + 0.06818 A + 0.01894 B + 0.2346 C + 0.000066 A.B – 0.002391 A.C - 0.002905 B.C + 0.000060 A.B.C (2)

| Table S2: Factorial experimental design matrix coded values and experimental results of the responses | | | | | |
| --- | --- | --- | --- | --- | --- |
|  | ***Coded values*** | | | ***Responses (%)*** | |
| *Run* | ***A (%)*** | ***B (°C)*** | ***C (h)*** | ***Y_1_*** | ***Y_2_*** |
| 1 | -1 | -1 | -1 | 88 | 30.11 |
| 2 | +1 | -1 | -1 | 29.2 | 36 |
| 3 | -1 | +1 | -1 | 80.031 | 30.68 |
| 4 | +1 | +1 | -1 | 26.801 | 37.45 |
| 5 | -1 | -1 | +1 | 80.88 | 32 |
| 6 | +1 | -1 | +1 | 27.6 | 39.12 |
| 7 | -1 | +1 | +1 | 79.71 | 29.65 |
| 8 | +1 | +1 | +1 | 23.83 | 43 |

| **Test on the normality of the residuals (Shapiro-Wilk) (Y_1_):** | | | |
| --- | --- | --- | --- |
| W | 0.914 |  |  |
| p-value (Two-tailed) | 0.382 |  |  |
| Alpha | 0.050 |  |  |
|  |  |  |  |

H0: The residuals follow a Normal distribution.

H1: The residuals do not follow a Normal distribution.

As the computed p-value is greater than the significance level alpha=0.05, one cannot reject the null hypothesis H0.

| **Test on the normality of the residuals (Shapiro-Wilk) (Y_2_):** | | | | |
| --- | --- | --- | --- | --- |
| W | 0.983 |  |  |  |
| p-value (Two-tailed) | 0.976 |  |  |  |
| Alpha | 0.050 |  |  |  |

H0: The residuals follow a Normal distribution.

H1: The residuals do not follow a Normal distribution.

As the computed p-value is greater than the significance level alpha=0.05, one cannot reject the null hypothesis H0.

| **Test for homoscedasticity of the residuals (Y_1_):** | | | |
| --- | --- | --- | --- |
| Factor | p-value |  |  |
| A | **0.047** |  |  |
| B | 0.341 |  |  |
| C | 0.326 |  |  |

The homogeneity of variances could be globally assumed for these factors.

The value in bold corresponds to tests where the null hypothesis is not accepted with a significance level alpha=0.05. However, we can see that the p-value of the factor A (0.047) is very close to the significance level alpha=0.05.

| **Test for homoscedasticity of the residuals (Y_2_):** | | |
| --- | --- | --- |
| Factor | p-value |  |
| A | 1.000 |  |
| B | 0.216 |  |
| C | 0.357 |  |

The homogeneity of variances could be assumed for these factors.

- **Analysis of the response Y_1_**

Analysis of variance (ANOVA) was carried out to justify the adequacy of the fitting model along with effects of their parameters (Table S2). A higher F-value indicates an adequacy of variation about its mean and a p-value (Prob. > F) less than 0.05 indicates the model is significant. From the ANOVA for the quadratic model of activation yield, the calculated F- and p-values were 123 and 0.039, respectively, which revealed the significance of the model (Table S2). On the other hand, a significant linear effect for the main factors has been revealed (P-value = 0.037), which indicates that the activation performance Y_1_ varies according to the linear terms.

In this model, the activating agent concentration (A) was a significant model term, whereas B, C, A-B, B-C, and A-C were negligible for the response. Hence, Eq. (2) can be used to study the effect of the activating agent concentration, activation temperature, and activation time on the activation yield.

The suitability of the model equations was evaluated using the correlation coefficients (R^2^) and R^2^ (adjusted), which are 0.998 and 0.9905 respectively. The proximity of R^2^ value to unity, indicates the suitability of the model equation and the good agreement between experimental data and the model prediction.

| Table S3. Analysis of Variance of activation yield Y_1_ | | | | | |
| --- | --- | --- | --- | --- | --- |
| Source | DF | Adj SS | Adj MS | F-Value | P-Value |
| Model | 6 | 6157.49 | 1026.25 | 123.00 | 0.039 |
| Linear | 3 | 6151.51 | 2050.50 | 245.76 | 0.037 |
| A | 1 | 6103.47 | 6103.47 | 731.51 | 0.024 |
| B | 1 | 29.53 | 29.53 | 3.54 | 0.311 |
| C | 1 | 18.51 | 18.51 | 2.22 | 0.376 |
| Interactions | 3 | 5.98 | 1.99 | 0.24 | 0.867 |
| A*B | 1 | 1.15 | 1.15 | 0.14 | 0.774 |
| A*C | 1 | 1.15 | 1.15 | 0.14 | 0.774 |
| B*C | 1 | 3.69 | 3.69 | 0.44 | 0.627 |
| Error | 1 | 8.34 |  |  |  |
| Total | 1 | 6165.3 |  |  |  |
| R^2^ = 0.9986; R^2^(Adj) = 0.9905 | | | | | |

On the other hand, the Pareto’s chart for the activation yield (Fig. S4) illustrates the statistical significance of this parameter. In this chart, each bar is proportional to the standardized effect presented in decreasing order of importance. The vertical red line (reference line) allows to identify the statistically significant effects at 95% confidence level. Therefore, according to the Pareto chart (Fig. S3), within the three studied factors (A: H_3_PO_4_ concentration, B: activation temperature, and C: the activation duration), only the activating agent concentration has a significant effect on the activation yield. Furthermore, interactions between these different factors did not reveal any significant effect on the activation yield (Y_1_).

**Fig. S3:** Pareto chart of the effects of factors on Y_1._ A: H_3_PO_4_ concentration, B: activation temperature, and C: the activation time.

- ***Response surface analysis***

To establish the optimal response values and operating conditions for the activation yield, contour and surface plots for factor interactions were constructed (Fig. S3). In a contour plot, the response surface is viewed as a 2D plane where all points that have the same response are connected to produce contour lines of constant responses. However, a surface plot generally displays a 3D view giving a clearer picture of the response. If the regression model (first-order model) contains only the main effects and no interaction effect, the fitted response surface will be a plane. If the model contains interaction effects, the contour lines will be curved and not straight. However, a second-order model will produce an elliptical contour [3].

In this study, the combination of the activating agent percentage with activation temperature (A-B) or with activation time (A-C) suggests that the higher the H_3_PO_4_ concentration, the higher the mass loss in the activation process (Fig. S4.a, b). From these, the highest values of activation yield (Y_1_ > 80%) are obtained when the three factors are set at low levels. Therefore, the optimal point and best yield are obtained under the following conditions:

- The percentage of activating agent: less than 22 % of H_3_PO_4_

- Activation temperature: two hours at 50°C.

Moreover, according to figures S4a, b and c we notice that the most intense factor on the yield is the factor of percentage of activating agent compared to the other two factors B and C (time and temperature of activation). And as we notice in Fig. 5.a and c, the areas of yield decrease are perpendicular to the axis (A) of percentage of activating agent, which shows that this factor is responsible for the yield decrease. The same trend was found by M. Loredo-Cancino et al [4], when determining the optimal conditions for activated carbon production from barley husks. Shows that when both temperature and impregnation rate are increased, the yield response decreases [5].

***
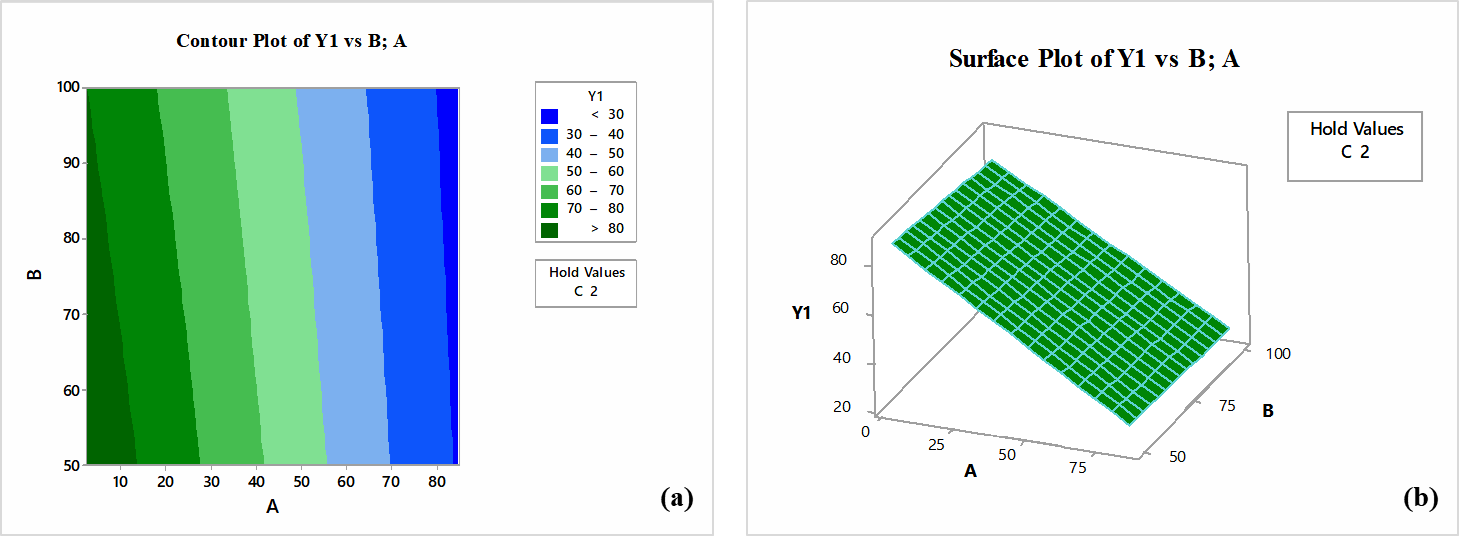
***

**Fig. S4. a:** Contour (a) and response surface (b) plots for the A-B interaction at C=2h on Y_1_.

***
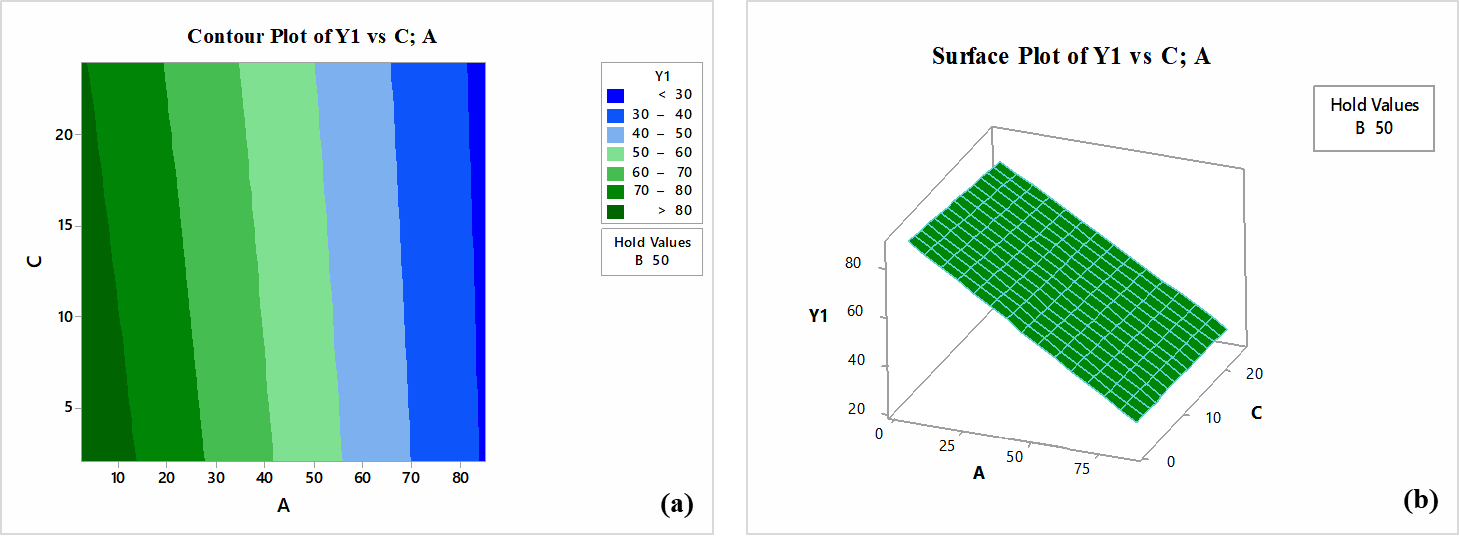
***

**Fig. S4. b:** Contour (a) and response surface (b) plots for the A-C interaction at B=50 °C on Y_1_.

***
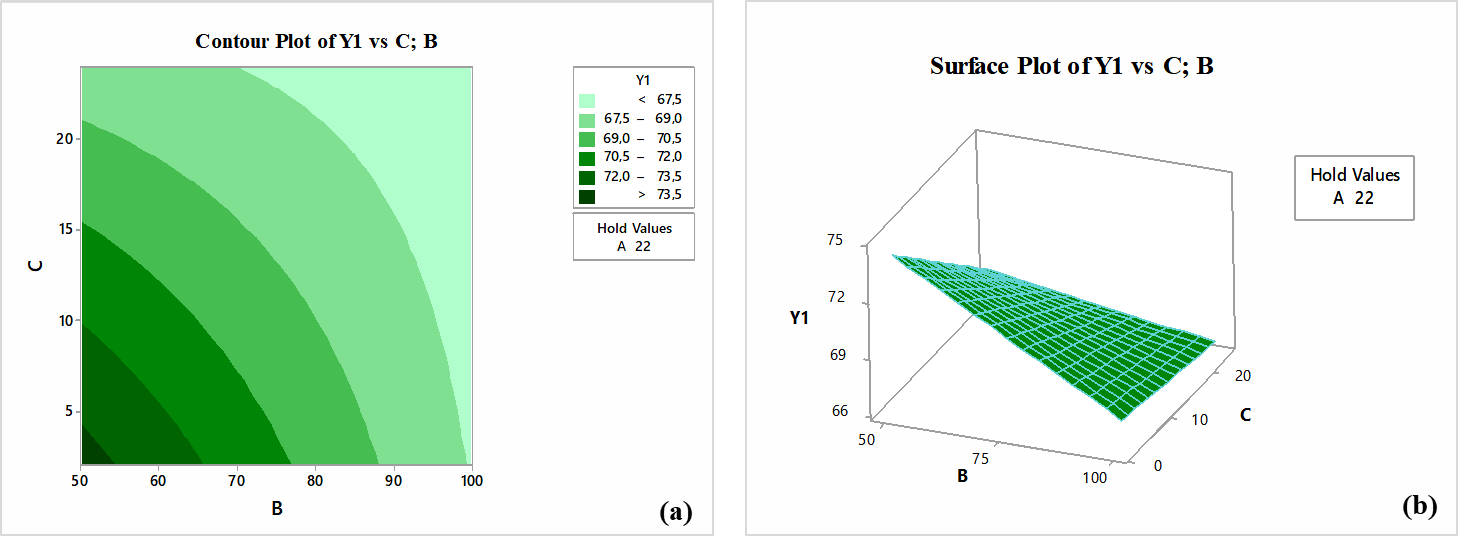
***

**Fig. S4. c:** Contour (a) and response surface (b) plots for the B-C interaction at A=22 % on Y_1_.

- **Analysis of the response Y_2_**

The analysis of variance (ANOVA) used to determine the significance of curvature in the response of pyrolysis yield Y_2_ is presented in Table S4. Based on the data, the P value=0.275 greater than 0.05 and F=7.38 lower, indicating that the model is not significant. On the other hand, the set of linear effects and double interactions of P-value indicates that there is no significant linear effect for the main factors. The coefficient of determination R^2^ and R^2^(adjusted) for pyrolysis yield were 0.9779 and 0.8205, respectively. This result indicates that the models could account for approximately 98% and 82% of the observed variability for the Y_2_ responses, respectively [4].

| Table S4. Analysis of Variance for pyrolysis yield Y_2_ | | | | | |
| --- | --- | --- | --- | --- | --- |
| Source | DF | Adj SS | Adj MS | F-Value | P-Value |
| Model | 6 | 164.984 | 27.497 | 7.38 | 0.275 |
| Linear | 3 | 151.403 | 50.468 | 13.54 | 0.197 |
| A | 1 | 138.112 | 138.112 | 37,06 | 0.104 |
| B | 1 | 1.674 | 1.674 | 0.45 | 0.624 |
| C | 1 | 11.616 | 11.616 | 3.12 | 0.328 |
| Interactions | 3 | 13.581 | 4.427 | 1.21 | 0.569 |
| A*B | 1 | 6.125 | 6.125 | 1.64 | 0.422 |
| A*C | 1 | 7.411 | 0.045 | 1.99 | 0.393 |
| B*C | 1 | 0.045 | 3.726 | 0.01 | 0.930 |
| Error | 1 | 3.72 |  |  |  |
| Total | 7 | 168.71 |  |  |  |
| R^2^ = 0.9779; R^2^(Adj) = 0.8205 | | | | | |

Regarding the pyrolysis yield (Y_2_), and from the results presented in the diagram of effects Fig. S5, highlighting that the three factors studied (A, B and C) has a non-significant influence on the pyrolysis yield Y_2_. But through the Pareto diagram we notice that the percentage of activating agent has an effect closer to the average effect of the factors. And the same for the interactions between the different factors studied has no significant effect on the yield of pyrolysis. This was demonstrated by the method 1 by the study of influence of different factors in the previous part Fig S4.b, which shows that the percentage of activating agent has a medium effect on the pyrolysis yield.

**Fig. S5:** Pareto chart of the effects of factors on Y_2_, A: H_3_PO_4_ concentration, B: activation temperature, and C: the activation time.

- ***Response surface analysis***

For graphical interpretation of interaction effects, the use of three-dimensional plots of the regression model is highly recommended [5-6]. Therefore, the 3D response contour and surface curves were plotted by the statistical model to understand the interaction of factors [7]. The model plot (Eq.2) facilitates examination of the effects of experimental factors on responses, 3D surface plots and contour plots between factors are shown in Fig S6.a, S6.b and S6.c.

Fig S6.a represented the effects of the percentage of agent (A) and the activation temperature (B) on the pyrolysis yield with the activation time set at low level (2h), the interaction between these factors generates an increase in the pyrolysis yield from 30.11% to 37.45%, that is to say leads to an increase in yield of about 7%. and the two-dimensional representation of the responses in terms of percentage of agent and activation time (C) shows that the A-C interactions between these two factors have no effect on the yield of pyrolysis Y_2_ (Fig. S6.b), generates an increase in yield from 30.11% to 39.12%.

Fig. S6.c shows the three-dimensional and contour response surface that were constructed to show the interaction effects of activation temperature (B) and activation time (C) on Y_2_ yield. For this plot, the percentage of activating agent was set at 22%. As can be seen in the figure that the interaction of the two B-C factors did not influence the pyrolysis yield, leading to an increase in yield of about 2%.


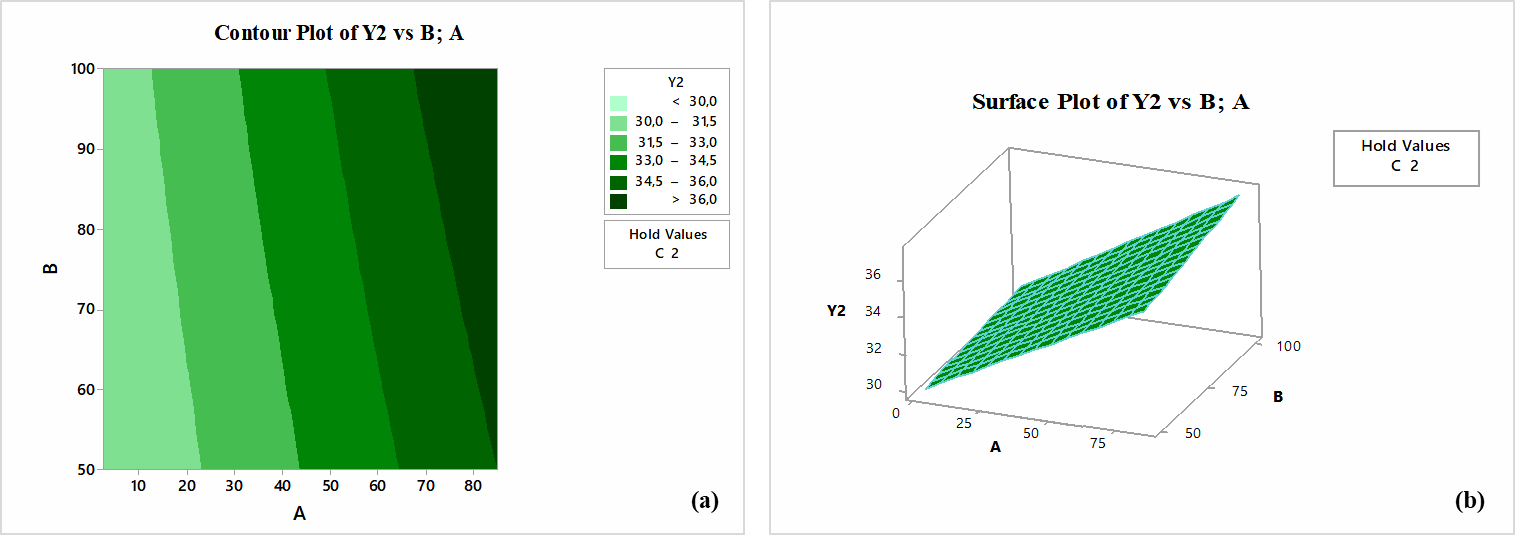


**Fig. S6. a:** Contour (a) and response surface (b) plots for the A-B interaction at C=2h on Y_2_.


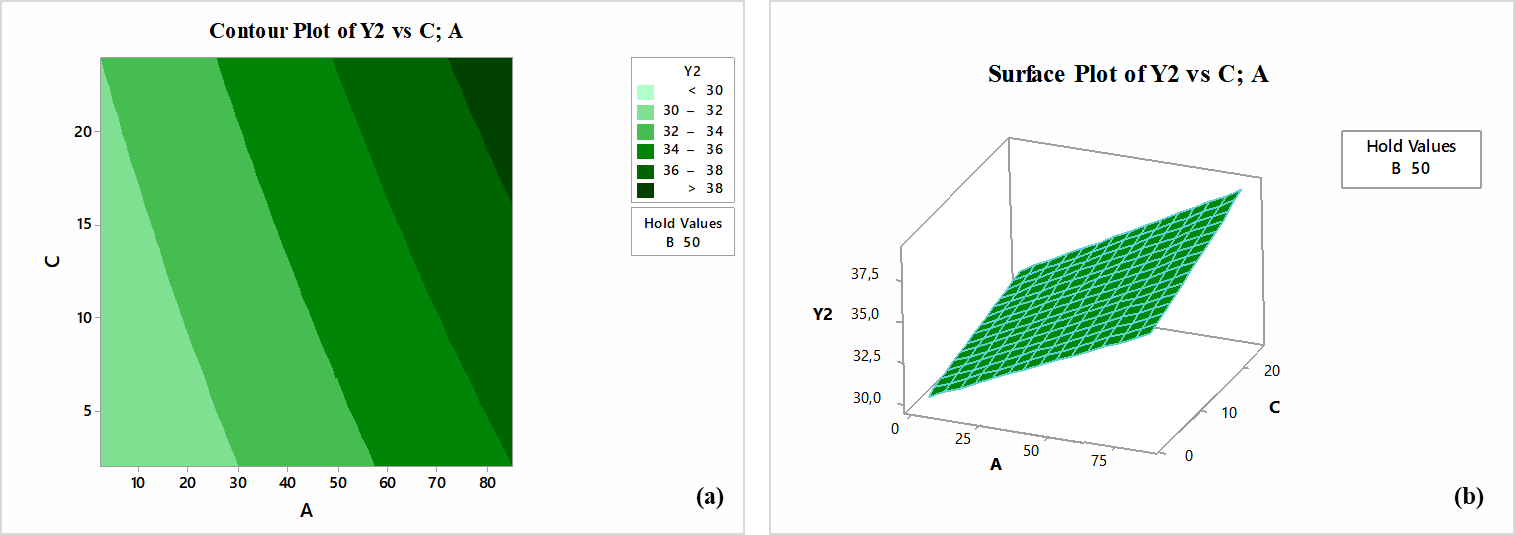


**Fig. S6. b:** Contour (a) and response surface (b) plots for the A-C interaction at B=50 °C on Y_2_.

***
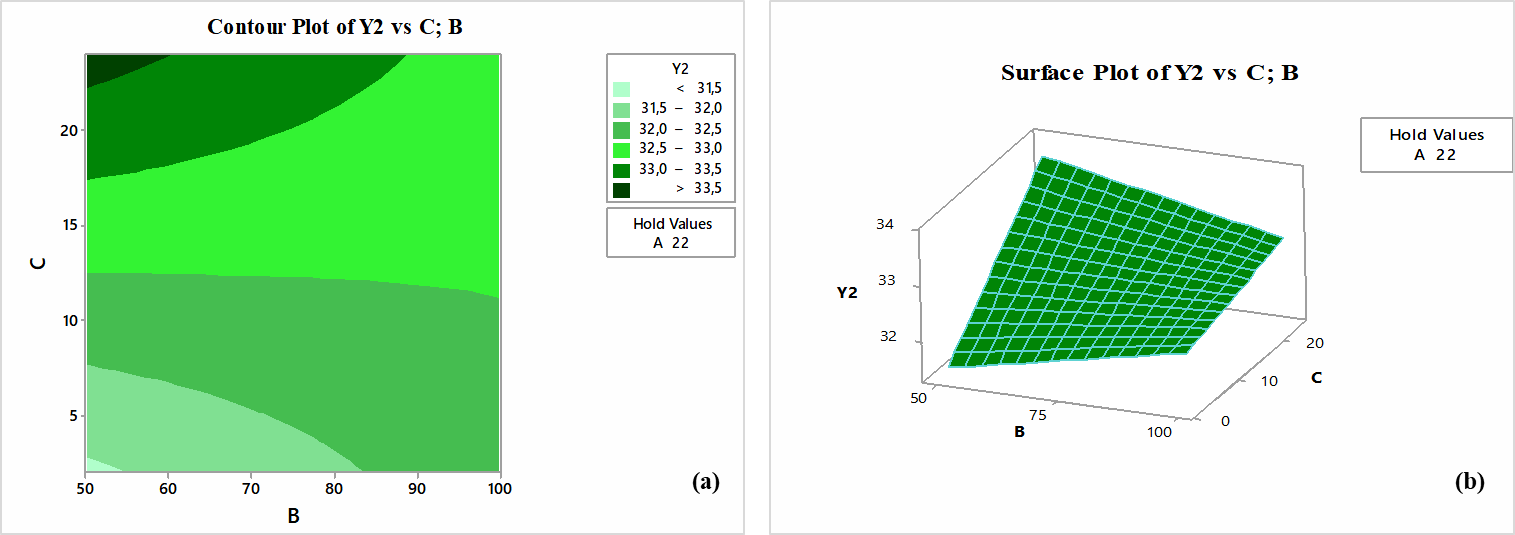
***

**Fig. S6. c:** Contour (a) and response surface (b) plots for the B-C interaction at A=22 % on Y_2_.

3. Conclusion.

As can be seen in, the experimental values for all responses of preparation conditions were in good agreement with the amounts predicted by the model. According to these results, the optimal point and best yield are obtained under the following conditions: Activation temperature is 50°C, (ii) activation time is 2h and, (iii) the percentage of activating agent around 22 % of H_3_PO_4_.

**References**

[1] Y.P. Huang, C.H. Hou, H.C. Hsi, and J.W. Wu., Optimization of highly microporous activated carbon preparation from Moso bamboo using central composite design approach, J. Taiwan Inst. Chem. Eng, 50 (2015) 266–275.

[2] M. Abbas, S. Kaddour, and M. Trari., Kinetic and equilibrium studies of cobalt adsorption on apricot stone activated carbon, J. Ind. Eng. Chem. 20 (2014) 745–751.

[3] Antony, J., Design of Experiments for Engineers and Scientists. Butterworth-Heinemann (Second Edition), 2014, New York.

[4] M. Loredo-Cancino, E. Soto-Regalado, F. J. Cerino-Córdova, R. B. García-Reyes, A. M. García-León, and M. T. Garza-González, Determining optimal conditions to produce activated carbon from barley husks using single or dual optimization, J. Environ. Manage. 125 (2013) 117–125.

[5] N. Aktaş, Optimization of biopolymerization rate by response surface methodology (RSM), Enzyme Microb. Technol*.* 37 (2005) 441–447.

[6] J. Antony, Design of Experiments for Engineers and Scientists. Butterworth-Heinemann (Second Edition), 2014, New York.

[7] A.A. Ahmad and B.H. Hameed, Effect of preparation conditions of activated carbon from bamboo waste for real textile wastewater, J. Hazard. Mater. 173 (2010) 487–493.
